# Circulating Extracellular Vesicles Carry Immune Regulatory miRNAs and Regulate Vaccine Efficacy and Local Inflammatory Response After Vaccination

**DOI:** 10.3389/fimmu.2021.685344

**Published:** 2021-06-15

**Authors:** Hiroyuki Oshiumi

**Affiliations:** Department of Immunology, Graduate School of Medical Sciences, Faculty of Life Sciences, Kumamoto University, Kumamoto, Japan

**Keywords:** microRNA, vaccine, extracellular vesicle, innate immunity, cytokine

## Abstract

Vaccination is the best prophylaxis for the prevention of infectious diseases, including coronavirus disease 2019. However, the efficacy of vaccines and onset of adverse reactions vary among individuals. Circulating extracellular vesicles (EVs) regulate the immune responses after vaccination by delivering microRNAs (miRNAs) to myeloid and lymphoid cells. Among these, miR-192 levels in serum EVs increase with aging, in an IL-6-dependent manner, reducing excessive IL-6 expression in aged mice, creating a negative feedback loop. Excessive IL-6 expression reduces vaccination efficacy in aged mice, while EV miR-192 improves efficacy in these aged mice as well, making this miRNA an interesting focus of study. miR-21 levels in serum EVs also increase with aging, and regulates the expression of IL-12 required for Th1 responses; therefore, EV miR-21 is expected to regulate vaccine efficacy. miR-451a, another important miRNA, is abundant in serum EVs and controls the expression of cytokines, such as type I interferon and IL-6. However, levels differ among individuals and correlate with local inflammatory symptoms experienced after a seasonal flu vaccination. These findings suggest the importance of EV miRNAs as a tool to improve vaccine efficacy and also as biomarkers to predict the immune response and adverse reactions after vaccinations.

## Introduction

Vaccines are the best prophylaxis for infectious disease prevention, including seasonal flu and coronavirus disease 2019 (COVID-19) (1, 2). Vaccines comprise specific antigens and adjuvants (3); several types of adjuvants, such as aluminum salts and monophosphoryl lipid A, are used in vaccines (4, 5). These induce pro-inflammatory cytokine expression, and activate dendritic cells and macrophages, leading to the priming of naïve T cells and provoking antigen-specific immune responses, including B-cell activation and antibody production (3). In addition to artificial compounds, components of viral particles also function as adjuvants, e.g., in the inactivated whole-virus influenza vaccine. Viral RNA within its viral particles that are recognized by Toll-like receptors determine the efficiency of vaccines (6); thus, adjuvant-induced innate immune responses are crucial for vaccine efficacy.

Studies have revealed that several microRNAs (miRNAs) regulate the innate immune responses (7–10). miRNAs are delivered from the host to donor cells by extracellular vesicles (EVs), such as exosomes and microvesicles (11–13). Exosomes are small vesicles, approximately 100 nm in diameter that express CD9, CD63, and CD81 proteins (14, 15), while microvesicles are > 100 nm in diameter (14, 16). Several miRNAs within EVs affect immune responses after vaccination.

## EV miR-192 Improves Age-Associated Decreases in Vaccine Efficacy

Aging affect the immune system (17, 18), and the efficacy of vaccines decreases with age (19). It is expected that aging would lead to immune dysfunction because of impaired B cell generation, a reduction in naïve T cells, a decreased ability of hematopoietic stem cells to replicate, and/or some other phenomena associated with age (20, 21). However, several studies have shown that chronic inflammatory responses increase with age, thereby decreasing vaccination efficacy (17, 22–24). For example, excessive TNF-α down-regulates CD28 expression on T cells (25), and high TNF-α levels lead to reduced B cell responses (26). These former studies suggest that excessive inflammation diminishes vaccine efficacy (27).

Decreased vaccine efficacy with aging has been observed in mouse animal models; mice aged 8–12 weeks are usually used for immune response analyses, and older mice, (1> year), exhibit lower vaccination efficacy than young mice (22, 28).

miR-192 is a miRNA induced by p53 that improves renal fibrosis in diabetic nephropathy patients (29, 30) and plays a role in several other diseases (31, 32). Recently, we found that miR-192 was an aging-associated miRNA and that EVs delivered miR-192 to macrophages, thereby reducing pro-inflammatory cytokine expression in the lungs (22). miR-192 targets ZEB2, MIP2α, TRIM25, IL-17RA, and Rictor mRNAs (**Table 1**): MIP2α is a chemokine that recruits neutrophils; TRIM25 is required for pro-inflammatory cytokine expression in response to influenza A virus RNAs (45, 46); and IL-17RA is crucial for pro-inflammatory cytokine expression in response to IL-17 (47). These targets might be involved in miR-192-mediated suppression of pro-inflammatory cytokine expression.

**Table 1:** Immune regulatory miRNAs and their targets.

miRNA	Target	Reference
miR-192	ZEB2	(30, 33)
	MIP2α (CXCL2)	(34)
	TRIM25	(35)
	IL-17RA	(36)
	Rictor	(37)
miR-21	IL-12p35	(38)
	PDCD4	(39)
	PTEN	(40)
miR-451	14-3-3ζ	(41, 42)
	CAB39	(43)
	IKK-β	(44)

**Figure 1:**
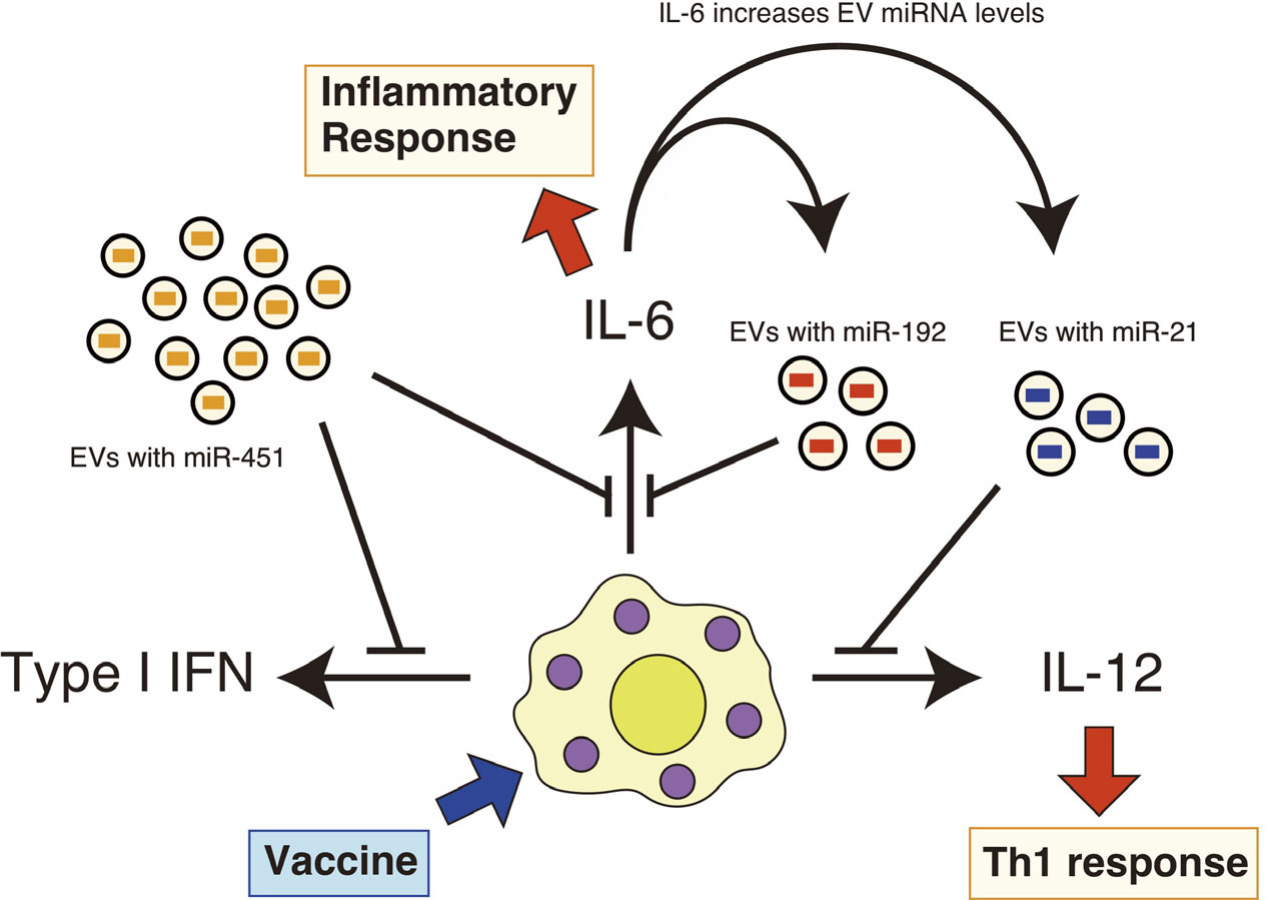
EV miRNAs regulate cytokine expression in response to vaccines. EVs deliver miR-451a, miR-192, and miR-21 to recipient cells, such as macrophages. Vaccine adjuvants then stimulate these macrophages and dendritic cells, resulting in the production of pro-inflammatory cytokines. miR-451a attenuates type I IFN and IL-6 expression in macrophages, and miR-192 reduces the expression of IL-6. miR-21 has the ability to attenuate the IL-12 expression. miR-192 and miR-21 levels in EVs increase with aging in an IL-6-dependent manner. Serum IL-6 levels are increased with aging, and thus constituting a miR-192-dependen negative feedback loop.

Additionally, we found that the expression of pro-inflammatory cytokines in the lung was prolonged in aged mice after intranasal administration of a whole-virus influenza vaccine (22). However, intranasal administration of EVs containing miR-192 mimic RNA reduced excessive pro-inflammatory cytokine expression, such as IL-6, and improved antigen-specific antibody levels after vaccination in aged mice (22). Since EV miR-192 levels increased in aged mice in an IL-6-dependent manner (22), it was expected that miR-192 would constitute a negative feedback loop to attenuate chronic inflammatory responses, resulting in improved immune responses and improved vaccination efficacy in elderly (**Figure 1**) (22).

## miR-21 Regulates Immune Responses After Vaccination

Serum IL-6 levels increase with aging in humans and mice (48), and miR-19b, miR-21, miR-181c, and miR-322 levels in serum EVs also increase with aging in an IL-6-dependent manner (22). Among those aging-associated miRNAs, miR-21 is known to regulate the immune responses following vaccination.

miR-21 negatively regulates the expression of IL-12p35, as well as IL-6, IL-8, TNF-α, and IL-1β (38, 49, 50). Although a contradicting report has shown the miR-21-augmented pro-inflammatory cytokine expression of IL-1β and IL-6 in RAW264.7 cells (51), Knockout (KO) studies have shown that miR-21 KO also increased the expression of these pro-inflammatory cytokines as well (52, 53). Therefore, EV miR-21 is expected to attenuate the expression of these pro-inflammatory cytokines (**Figure 1**). miR-21 targets mRNAs of IL-12p35, PDCD4, and PTEN (**Table 1**): although PDCD4 promotes pro-inflammatory cytokine expression (54), PTEN reduces pro-inflammatory cytokine expression (40), and these mechanisms might underlie the apparent contradictions.

miR-21 levels affect the efficacy of a live-attenuated vaccine of Leishmania, *LdCen^-/-^* (55). IL-12 is an essential cytokine for Th1 response to Leishmania; therefore, miR-21 decreased Th1 immunity, thereby affecting the efficacy of this live-attenuated vaccine (55). Interestingly, miR-21-containing exosomes derived from dendritic cells regulate CD4^+^ T cell proliferation (56); thus, it is expected that serum EV miR-21 levels would affect innate and adaptive immune responses following vaccination. Further investigation is required to determine the role of EV miR-21 in the immune response after vaccination.

## miRNA-451a Is Associated With Local Inflammatory Responses After Vaccination

Innate immunity itself is required to initiate the adaptive immune responses, whereas it leads to a local inflammatory response. Upon stimulation from adjuvants, IL-6, TNF-α, and IL-1β are produced from macrophages and increase vascular permeability, allowing the flow of red and white blood cells as well as plasma with small molecules, resulting in local swelling, pain, and redness (57–60). Additionally, circulation of these cytokines in the blood flow causes prostaglandin E2 (PGE2) production in the hypothalamus, leading to fever (61). Excessive innate immune responses are harmful to the host: hypermorphic mutations in the genes involved in innate immune responses cause autoimmune disorders (62).

miR-451a attenuates pro-inflammatory cytokine expression (41), as it targets 14-3-3ζ (**Table 1**), which controls the activities of FOXO3 and ZFP36 (41). FOXO3 is an inhibitory transcription factor for cytokine expression (63), and ZFP36 can bind to AU-rich elements of the untranslated mRNA regions, thus destabilizing cytokine mRNAs (41). In addition to 14-3-3ζ, IKK-β and CAB39 are also targeted by miR-451a (**Table 1**): IKK-β plays a crucial role in NF-κB activation (64). These mechanisms are expected to underlie miR-451a-mediated suppression of pro-inflammatory cytokine expression.

miR-451a is efficiently sorted into EVs in several cell types (65, 66); therefore, miR-451a levels in serum EVs are very high (67, 68), and its intracellular levels are relatively low (65, 69). We found that EV miR-451a levels in the serum of a culture medium correlated with intracellular miR-451a levels, a few days after incubation of macrophages with a serum-containing medium, because EVs deliver miR-451a to macrophages (69). Therefore, EVs miR-451a levels in serum of culture medium correlated with expression levels of cytokines, such as type I IFN and IL-6, in macrophages stimulated with influenza A virus vaccines (69). This correlation was also observed in several other miRNAs (69).

In the short term, miR-451a levels in human sera are relatively stable, with few changes of more than two-fold in a week (69); however, levels gradually fluctuate and, in some cases, change by more than 10-fold during a year (69). IL-6 is a pro-inflammatory cytokine regulated by miR-451a that causes inflammatory responses, including vesicular permeability (41). Our clinical study showed that miR-451a levels in serum EVs before vaccination correlated with the occurrence of local inflammatory symptoms observed after a seasonal flu vaccination (68). Several other EV miRNAs were also associated with local inflammatory responses (68). Christian LM et al. have reported the correlation of local inflammatory symptoms with the expression of pro-inflammatory cytokines after vaccination (70). These observations imply that circulating EV miRNAs regulate local cytokine expression and inflammatory responses after vaccination. However, it is still possible that the levels of miR-451a and other immune regulatory miRNA reflect a physical condition that affects the inflammatory responses. Further studies are required to determine mechanism underlying the correlation between miR-451a levels in circulating EVs and immune responses after vaccination.

## Discussion

Vaccine efficacy varies among individuals, and vaccine-related adverse reactions occur only in certain cases. Environmental factors affect immune responses (71, 72) and are expected to cause differences in vaccine efficacy and the onset of adverse reactions. Increasing evidence has shown that circulating EV miRNAs affect immune responses after vaccination, and miRNA levels vary among healthy individuals. For example, excessive glucose uptakes and several diseases are reported to affect miR-451a levels (73–76). Some of the environmental factors that affect EV miRNA levels might regulate vaccine-induced immune responses; hence, studies of the environmental factors affecting circulating EV miRNA levels are important in identifying environmental factors affecting immune responses after vaccination.

Recent studies have identified the significant potential of serum miRNAs as biomarkers for cancer, diabetes, Alzheimer′s disease, allergic inflammatory disease, rheumatoid arthritis, etc. (77–80). Thus, since serum EV miRNAs affect the immune responses following vaccination, they can potentially be used as biomarkers to predict vaccine efficacy and adverse reactions. Similarly, if a vaccination is predicted to be ineffective for a person, improvement of the efficacy by an additional vaccination is possible. In the case of the recent COVID-19 outbreak, herd immunity will be crucial in eradicating the pandemic (81). Thus, prediction of the efficacy and follow-up vaccination requirements might help achieve effective herd immunity against COVID-19 efficiently. COVID-19 vaccination is progressing all over the world and, thus cohort studies investigating the association of EV miRNAs with antibody production or memory cell generation after vaccination would reveal the potential of EV miRNAs as biomarkers.

Vaccination efficacy decreases with age, therefore, miRNAs that affect or improve immune responses of the elderly would also help improve the vaccination efficacy. Indeed, miR-192 in EVs improves the efficacy of vaccination in aged mice. Recent approaches have tested the engineering of exosomes for delivering therapeutic proteins and nucleic acids, as well as miRNAs (82). Thus, it is expected that vaccines containing EV miR-192 would be useful for vaccinating the elderly. In addition to miR-192, miR-451a could improve vaccines. miR-451a levels in EVs were negatively correlated with inflammatory responses at the vaccination site and reduced pro-inflammatory cytokine expression (68, 69); therefore, the addition of EV miR-45 to a vaccine might improve excessive inflammatory symptoms, such as pain, swelling, and redness, without reducing efficacy. EVs containing immune regulatory miRNAs could be useful tools to improve vaccine efficacy and to reduce adverse reactions.

Although both exosomes and microvesicles deliver miRNAs, there are functional differences between them; for example, miR-150 is efficiently sorted into exosomes but not microvesicles (83). Furthermore, EVs can be classified into several types, and each type contains distinct components (84). It is therefore possible that specific EVs affect the immune response after vaccination. Further studies are required to reveal the role of EVs in regulating immune responses after vaccination.

## Author Contributions

The author confirms being the sole contributor of this work and has approved it for publication.

## Funding

This work was supported in part by Grants-in-Aid from the Japan Agency for Medical Research Development (AMED) and Society of the Promotion of Sciences (JSPS).

## Conflict of Interest

The author declares that the research was conducted in the absence of any commercial or financial relationships that could be construed as a potential conflict of interest.
